# Leukocyte telomere length and risk of coronary heart disease and stroke mortality: prospective evidence from a Russian cohort

**DOI:** 10.1038/s41598-018-35122-y

**Published:** 2018-11-09

**Authors:** Denes Stefler, Sofia Malyutina, Vladimir Maximov, Pavel Orlov, Dinara Ivanoschuk, Yury Nikitin, Valery Gafarov, Andrey Ryabikov, Mikhail Voevoda, Martin Bobak, Michael V Holmes

**Affiliations:** 1 Department of Epidemiology and Public Health, University College London, London, United Kingdom; 2 Research Institute of Internal and Preventive Medicine – Branch of IC&G SB RAS, Novosibirsk, Russia; 3 Medical Research Council Population Health Research Unit at the University of Oxford, Oxford, UK; 4 Clinical Trial Service Unit & Epidemiological Studies Unit (CTSU), Nuffield Department of Population Health, University of Oxford, Richard Doll Building, Old Road Campus, Roosevelt Drive, Oxford, OX3 7LF UK; 5 National Institute for Health Research Oxford Biomedical Research Centre, Oxford University Hospital, Oxford, UK

## Abstract

Previous studies suggest that reduced leukocyte telomere length (LTL) is related to higher risk of mortality and several chronic conditions, including coronary heart disease (CHD) and stroke. However, the consistency of this association differs across populations. We investigated the relationship of LTL with CHD, stroke and all-cause mortality together with non-fatal CHD and stroke events in a Russian cohort with a mean age of 58 years at baseline. Data from 1,144 individuals in the Russian subset of the Health Alcohol and Psychosocial Factors in Eastern Europe (HAPIEE) cohort study were used. The associations between LTL at baseline and fatal/non-fatal outcomes during 12 years of follow-up were assessed using multivariable Cox regression models, which yielded adjusted hazard ratios (HR). Compared to individuals in the shortest tertile, those in the longest tertile of LTL had a 42% lower risk of death from all-causes (HR 0.58; 95% CI: 0.39–0.88) and 58% lower risk of death from CHD (HR 0.42; 95%CI: 0.19–0.97). Similar patterns of association were identified for non-fatal and combined fatal/non-fatal CHD and stroke events but the associations were weaker. Consistent with results of previous studies in Western populations, this cohort of elderly Russian adults found an inverse association between LTL and CHD and all-cause mortality. These findings reinforce the hypothesis that LTL may play (or be a marker of) an aetiological role in human health across diverse populations.

## Introduction

Telomeres are repetitive DNA sequences located at the ends of each chromosome. Telomeres become shorter after every cell division and as such their length is considered to be a marker of biological ageing^1,2^. Consistent with this hypothesis, reduced leukocyte telomere length (LTL) and a higher rate of its attrition have been reported to associate with various chronic diseases, including cardiovascular disease (CVD), diabetes, cancer or Alzheimer`s disease^3–6^.

Most previous studies have reported inverse associations between LTL and mortality^7–10^. One of the largest was carried out on nearly 65,000 individuals on a Danish general population sample, which found a 40% difference in the risk of death between participants in the shortest and longest telomere length deciles^8^. However, the available evidence is not entirely consistent, with several studies reporting null findings, particularly those which examine older individuals above the age of 70 years^11,12^. Two recent studies from the US also found that the association of LTL with mortality differed by ethnicity^9,13^. Although their findings were conflicting, with one suggesting stronger and the other weaker association between LTL and cardiovascular mortality among African Americans compared to White Europeans, this observation emphasises the importance to explore the association of LTL with disease endpoints in diverse populations.

CVD is the leading cause of mortality worldwide, with coronary heart disease (CHD) and stroke estimated to be responsible for more than 15 million deaths in 2015^14^. Although age-standardised mortality rates due to CVD has been decreasing in most global regions, the absolute number of deaths from vascular causes has increased globally. In many Eastern European states, particularly in Russia, CVD mortality increased sharply in the mid-1990s, and even today, more people die of CVD than from all other causes combined^15^.

To date, studies of the relationship between LTL and mortality or CHD/stroke risk have been conducted in North America, Western Europe or Asian populations; so far there has been no such study in Eastern Europe. In this report, we investigated the association of LTL with all-cause, CHD and stroke mortality, as well as with non-fatal CHD/stroke events, in a Russian population sample using data from a prospective cohort study with 12 years of follow-up.

## Methods

### Study population and analytical sample

We conducted a prospective analysis using data from the Russian subset of the Health Alcohol and Psychosocial Factors in Eastern Europe (HAPIEE) cohort study. At baseline, the study recruited men and women aged 45–70 years in two districts of the city of Novosibirsk^16^. From the original 9360 participants (response rate 61%), 1144 individuals were randomly selected at baseline for the purpose of more intensive phenotyping. LTL was measured in this subsample.

All participants provided informed consent and study protocols were approved by ethical committees at University College London and Novosibirsk State Medical University. The study was conducted in accordance with the relevant ethical guidelines and regulations.

### Data collection

Baseline data collection of the HAPIEE study took place between 2003 and 2005. Participants filled in a comprehensive questionnaire and provided information on their socio-economic circumstances, lifestyle habits and health. They also underwent medical examination and provided venous blood samples.

Genomic DNA was isolated from the randomly selected 1144 blood samples by phenol-chloroform extraction, and stored at minus 70°C until further laboratory analysis. Telomere length analysis was performed using quantitative real-time PCR-based method^17,18^. As previously described^19^, quantitative reactions were conducted separately for telomeres and the hemoglobin gene in pairs of 96-well plates in corresponding wells. Each plate housed a series of DNA dilutions (0.5, 1, 2, 5, 10, 20, and 30 ng) which were utilized for the construction of a standard curve and quantitation of each sample. Each reaction was done with 10 ng of DNA. The composition of the reaction mixture for telomere analysis included 270 nM tel1b primer (5′-CGGTTT(GTTTGG)5GTT-3′), 900 nM tel2b primer (5′-GGCTTG(CCTTAC)5CCT-3′), 0.2X SYBR Green I, 5 mM dithiothreitol, 1% dimethylsulfoxide, 0.2 mM each dNTP, 1.5 mM MgCl_2_, and 1.25 U of DNA polymerase. The reaction volume was 15 μL. The PCR program was conducted as follows: a predenaturation step for 10 min at 95°C followed by 25 cycles: denaturation at 95°C for 15 s, annealing at 54°C for 30 s, and elongation at 72°C for 90 s. The reaction mixture for the β hemoglobin gene contained 300 nm Hbg1 primer (5′-GCTTCTGACACAACTGTGTTCACTAGC-3′), Hgb2 primer (5′-CACCAACTTCATCCACGTTCACC-3′), 0.2X SYBR Green I, 5 mM dithiothreitol, 1% dimethylsulfoxide, 0.2 mM each dNTP, 1.5 mM MgCl2, and 1.25 U of DNA polymerase. The PCR program began with a predenaturation step for 3 min at 95°C followed by 25 cycles: 95°C for 15 s, 58°C for 20 s, and 72°C for 20 s. Both reactions were carried out in a StepOnePlus™ Real-Time PCR System (Applied Biosystems, Thermo Fisher Scientific Inc., USA). Calculations were performed with the standard cycler software. Single-copy gene (T:S) ratio were done to obtain the relative LTL. If the standard deviation in three replications exceeded 0.5, the sample was rejected. Each plate housed three control samples with normal LTL and one with short. Relative signal intensities from control samples were tested to ensure the comparability of the plates.

As the applied DNA extraction method can affect the LTL results due to contamination^20^, the protocol included procedures of cleaning from protein and other organic substances. For quality control, we assessed the quantity and concentration of DNA using the Microplate Spectrophotometer Epoch (BioTek, USA). To assess the contamination by protein or other organic substances, 260/280 nm and 260/230 nm wave length concentration indexes were used, respectively. Quality limits of index 260/280>1.8 and index 260/230>2.0 indicated DNA which was not acceptable for analysis. Only 1% of the samples was excluded from the analysis due to this criterion. For these cases, DNA extraction was performed from another aliquot if they were available. To ensure the stability and consistency of the procedures all samples were analysed with the same DNA extraction method and laboratory equipment.

Participants were followed-up for mortality and non-fatal cardiovascular outcomes. Data on all-cause and cause-specific mortality were collected at the Research Institute of Internal and Preventive Medicine using a number of sources, including the Population Registration Bureau (ZAGS) and the Novosibirsk Office of the State Statistical Bureau (Rosstat). Regarding all-cause mortality, the available information was also updated when subjects were invited to participate in subsequent waves of the study, for example by speaking to relatives of deceased study participants. For cause-specific mortality, cause of death was available only for CHD and stroke. This is because access to death registration data was not available between 2011 and 2015 due to administrative reasons; however, CHD [ICD-10: I20-I25] and stroke deaths [ICD-10: I60-I69] could be identified via the myocardial infarction (MI) and stroke registers (described below). Non-fatal CHD and stroke events were primarily identified through the Research Institute of Internal and Preventive Medicine’s registers of MI and stroke, originally established in the frame of the WHO MONICA project^21^, using medical records and hospital discharge reports. Non-fatal CHD and stroke events were additionally confirmed and identified by self-report in subsequent waves of data collection. The mean follow-up time for mortality and non-fatal cardiovascular events was 12 years.

### Statistical analysis

The associations between LTL and fatal/non-fatal end-points were assessed using Cox regression analysis. LTL was analysed as both a categorical and continuous variable. The categorical variable was derived as the tertiles of LTL; the group with the shortest LTL was used as the reference category. When LTL was used as a continuous variable, the hazard ratio of mortality or CVD events was modelled per one standard deviation (SD) higher LTL (in our dataset, 1 SD was equal to 0.41 kbp). Proportionality assumptions were tested with Schoenfeld residuals.

We examined the associations in two multivariable adjusted models. In model 1, hazard ratios were adjusted for age and sex as these are the two most common confounders. In model 2, hazard ratios were further adjusted for those socio-demographic, lifestyle and metabolic factors which are often associated with both LTL and mortality outcomes^22,23^. These were: smoking (never; ex-; regular smokers), alcohol intake (abstainers; moderate drinkers – intake less than 15 g/day for females or 30 g/day for males; heavy drinkers – intake more than 15 g/day for females or 30 g/day for males), education attainment (primary or less; vocational; secondary; higher degree), marital status (living with or without partner), body mass index, systolic blood pressure, serum cholesterol concentration and self-reported history of prior CVD.

All statistical analysis was carried out with the statistical software Stata 13.1 *(StataCorp, TX, USA)*.

## Results

Table 1 shows the distribution of covariates across LTL tertiles at baseline. Individuals with longer LTL were younger and they were more likely to be women and non-smokers. Other than age and sex, none of the other baseline cardiovascular risk factors showed particularly convincing associations with LTL. Distribution of covariates across LTL tertiles were mostly similar to the pooled results when men and women were examined separately, however, the inverse association with blood pressure was observed only in females (Supplementary Tables S1 and S2).

**Table 1:** Distribution of covariates across leucocyte telomere length tertiles at baseline in the Russian arm of the HAPIEE study.

Covariates	Leucocyte telomere length tertiles			p-value^a^
	Shortest LTL	Middle LTL	Longest LTL	
	n = 382	n = 381	n = 381	
	(range: 0.14–1.15 kbp)	(range: 1.15–1.50 kbp)	(range: 1.50–4.08 kbp)	
Age in years (mean, SD)	58.4 (6.9)	57.9 (6.8)	56.2 (6.8)	<0.001
Females (%)	44.0	58.8	57.7	<0.001
Current smokers (%)	30.9	21.3	23.4	0.006
Heavy drinkers (%)	11.0	7.6	9.2	0.235
Married (%)	79.1	73.0	75.9	0.144
University education (%)	34.8	27.8	36.5	0.101
Systolic blood pressure (mean, SD)	140.7 (23.2)	141.9 (24.9)	137.8 (23.1)	0.050
Body mass index (mean, SD)	28.1 (4.7)	28.4 (5.1)	28.4 (5.3)	0.575
Total cholesterol cc. (mean, SD)	6.4 (1.2)	6.5 (1.3)	6.4 (1.1)	0.544
Self-reported history of prior CVD (%)	21.2	21.0	19.2	0.743

SD – standard deviation; LTL – leukocyte telomere length; CVD – cardiovascular disease.

^a^ANOVA or Chi-square test.

During 12 years of follow-up, 149 participants died, including 58 due to CHD or stroke and 89 due to non-vascular causes. Composite fatal and non-fatal CHD and stroke end points included 182 events (124 non-fatal). Individuals in the longest LTL tertile had a lower risk of death from any cause as compared to those in the shortest LTL tertile (HR 0.57, 95%CI 0.38–0.85; P=0.003 for trend across the three tertile groups), and the association was similar when LTL was modelled as continuous variable (HR 0.81, 95%CI 0.68–0.97 per 1-SD longer LTL; P=0.021) (Table 2). Similarly, longer LTL was associated with lower risk of death from CHD (HR 0.44, 95% CI 0.20–0.99 comparing longest to shortest LTL tertile; P=0.043 for trend across the three tertiles), with the equivalent estimate for death from CHD being HR 0.72 (95%CI: 0.52–1.00; P=0.047 per 1-SD longer LTL). Combining CHD and stroke deaths together, the estimates were directionally consistent, but the 95%CI were wider (Table 2). Regarding non-cardiovascular deaths, the association was not linear, as the lowest risk was observed in the middle tertile of LTL. This pattern, which also had an impact on the linearity of the association with all-cause mortality, may reflect the heterogeneous nature of this outcome (which includes, for example, cancer and external causes of deaths). All examined associations remained robust to multivariable adjustment (model 2 in Table 2).

**Table 2:** Relationship between leucocyte telomere length tertiles and mortality outcomes in the Russian arm of the HAPIEE study.

Cause of death	n death	model	Leucocyte telomere length tertiles						Per 1 SD increase in telomere length		
			Shortest LTL	Middle LTL		Longest LTL		p-value for trend			
			HR	HR	(95% CI)	HR	(95% CI)		HR	(95%CI)	p-value
Any cause	149	model 1	1.00 (ref)	0.61	(0.41–0.90)	0.57	(0.38–0.85)	0.003	0.81	(0.68–0.97)	0.021
		model 2	1.00 (ref)	0.57	(0.38–0.84)	0.58	(0.39–0.88)	0.004	0.82	(0.69–0.98)	0.030
CHD (ICD-10: I20-I25)	46	model 1	1.00 (ref)	0.75	(0.38–1.45)	0.44	(0.20–0.99)	0.043	0.72	(0.52–1.00)	0.047
		model 2	1.00 (ref)	0.68	(0.34–1.33)	0.42	(0.19–0.97)	0.035	0.72	(0.51–1.00)	0.050
CHD or Stroke (ICD-10: I20-I25 or I60-I69)	58	model 1	1.00 (ref)	0.98	(0.55–1.75)	0.57	(0.28–1.15)	0.141	0.79	(0.60–1.05)	0.108
		model 2	1.00 (ref)	0.89	(0.49–1.61)	0.56	(0.27–1.15)	0.128	0.80	(0.60–1.07)	0.129
Non-CVD causes (ICD-10 codes other than I00-I99)	89	model 1	1.00 (ref)	0.44	(0.25–0.76)	0.58	(0.35–0.96)	0.016	0.85	(0.68–1.06)	0.156
		model 2	1.00 (ref)	0.41	(0.24–0.71)	0.62	(0.37–1.03)	0.025	0.86	(0.69–1.08)	0.202

LTL – leukocyte telomere length; HR – hazard ratio; CI – confidence interval; SD – standard deviation; CHD – coronary heart disease; ICD-10–10^th^ revision of the International Classification of Diseases.

model 1: adjusted for age and sex.

model 2: adjusted for age, sex, smoking, alcohol, education, marital status, body mass index, systolic blood pressure, total cholesterol cc. and self-reported history of prior CVD.

The association of the combined fatal and non-fatal CHD and stroke events with LTL is shown in Table 3. In keeping with the associations with vascular mortality, the point estimates of the effect estimates were consistent with a potential underlying inverse relationship between LTL and fatal/nonfatal CHD and stroke; however, the associations were weak and the 95%CI included the null. For example, the per-SD longer LTL association with fatal/nonfatal CHD was HR 0.83 (95%CI: 0.69–1.00; P=0.051). When non-fatal outcomes were examined separately, the strength of most associations was found to be even weaker (Supplementary Table S3).

**Table 3:** Relationship between leucocyte telomere length tertiles and combined fatal or non-fatal CHD and stroke events in Russian arm of the HAPIEE study.

Cardiovascular outcome (fatal or non-fatal)	n event	model	Leucocyte telomere length tertiles						Per 1 SD increase in telomere length		
			Shortest LTL	Middle LTL		Longest LTL		p-value for trend			
			HR	HR	(95% CI)	HR	(95% CI)		HR	(95%CI)	p-value
CHD	134	model 1	1.00 (ref)	0.80	(0.53–1.20)	0.75	(0.49–1.14)	0.168	0.83	(0.69–1.00)	0.051
		model 2	1.00 (ref)	0.73	(0.48–1.09)	0.77	(0.51–1.18)	0.203	0.85	(0.70–1.02)	0.077
Stroke	56	model 1	1.00 (ref)	0.75	(0.40–1.41)	0.77	(0.40–1.46)	0.397	0.88	(0.67–1.18)	0.400
		model 2	1.00 (ref)	0.71	(0.38–1.34)	0.74	(0.39–1.43)	0.349	0.88	(0.66–1.17)	0.368
CHD or stroke	182	model 1	1.00 (ref)	0.83	(0.58–1.17)	0.81	(0.56–1.16)	0.228	0.87	(0.75–1.02)	0.087
		model 2	1.00 (ref)	0.75	(0.53–1.07)	0.81	(0.56–1.16)	0.217	0.88	(0.75–1.03)	0.100

LTL – leukocyte telomere length; HR – hazard ratio; CI – confidence interval; SD – standard deviation; CHD – coronary heart disease.

model 1: adjusted for age and sex.

model 2: adjusted for age, sex, smoking, alcohol, education, marital status, body mass index, systolic blood pressure, total cholesterol cc. and self-reported history of prior CVD.

## Discussion

In this prospective analysis of Russian adults, we found longer LTL to be associated with a lower risk of fatal CHD and all-cause mortality. We identified directionally consistent inverse relationships of LTL with risk of combined fatal/non-fatal CHD and stroke outcomes, however these associations was weaker and largely driven by fatal outcomes.

This is the first study to explore the relationship of LTL and risk of cause-specific mortality in an Eastern European prospective cohort. Our findings are consistent with previous reports and highlight the role that LTL may play in disease risk. Our findings, that subjects who have longer LTL have a 56% lower risk of fatal CHD is in keeping with a meta-analysis of 24 studies including 8,400 cases of CHD, of which 248 were fatal, and which identified a 48% lower risk of fatal CHD for a similar comparison^3^. Similar to this previously reported study, the relationships we report were robust to inclusion of conventional cardiovascular risk factors.

The exact mechanisms linking telomere length with mortality or chronic diseases are not entirely clear, and a number of potential plausible explanations exist. Some authors suggest the possibility of a direct effect, potentially through the role of cellular senescence in atherosclerosis^24^. Indeed, a Mendelian randomization study suggests that LTL may be causal risk factor for CHD^25^, however such studies need to be interpreted with caution, especially when genetic variants may be affected by pleiotropy^26^. In contrast to being causally implicated, it is also possible that LTL represents a marker of cardiovascular ageing because unhealthy lifestyle (e.g. smoking, alcohol, poor diet) and the consequent oxidative stress affect both LTL and the risk of CVD independently^27^. A recent review provides several reasons for a cautionary interpretation of whether LTL represents a causal risk factor for ageing^28^. For example, it is argued that the inverse association of LTL with mortality attenuates with advancing age^29^. While it may be difficult to compare estimates between studies (given the opportunity for ecological confounding^30^), our study, with a mean age of ~60 and followed up for 12 years, provides new evidence that in East European populations, LTL does associate with death in older age. We stress, however, that our findings are observational in nature, and cannot be used to deduce cause and effect.

Our findings has strengths including studying an under-represented population, a prospective study design, and cause-specific mortality from Russian registries. Our study also has limitations including a rather small sample size and potential for misclassification of the non-fatal outcomes (which is the most likely reason for the weaker associations with LTL observed for non-fatal disease). Our inability to separate out non-vascular disease into specific causes means we are unable to tease out why the relationship of LTL with non-vascular death appeared U-shaped – this likely reflects differing effects on discrete causes of death, and accounts for a similar plateauing of the association of all-cause mortality comparing those with intermediate and long LTL to those with short LTL.

In conclusion, our study identifies longer LTL to be associated with substantial reductions in risk of CHD and all-cause mortality in a prospective cohort of elderly Russians. This highlights the need for further investigations to elucidate the mechanism by with LTL may impact human health.

## Electronic supplementary material

Supplementary tables

## Data Availability

Availability of data from the HAPIEE study is restricted due to legal reasons. Further information and access can be requested by contacting the principle investigator, Professor Martin Bobak (m.bobak@ucl.ac.uk) who will seek approval by the HAPIEE Study Steering Committee and the Research Ethics Committee at UCL and participating centres.
