# Supplementary material for: Leukocyte telomere length and risk of coronary heart disease and stroke mortality: prospective evidence from a Russian cohort
Source: Sci Rep. 2018 Nov 9;8:16627. doi: 10.1038/s41598-018-35122-y (PMC6226519; doi:10.1038/s41598-018-35122-y)
Supplement: Supplementary file 1 — Supplementary tables [file 41598_2018_35122_MOESM1_ESM.pdf]

SUPPLEMENTARY MATERIAL

Leukocyte telomere length and risk of coronary heart disease and stroke mortality: prospective evidence from a Russian cohort

Stefler D, Malyutina S, Maximov V, Orlov P, Ivanoschuk D, Nikitin Y, Gafarov V, Ryabikov A, Voevoda M, Bobak M, Holmes M

Table S1 (supplementary material). Distribution of covariates across leucocyte telomere length tertiles among men

Covariates	Leucocyte telomere length tertiles						p-value[a]
	Shortest LTL n=178 (range: 0.14-1.10 kbp)		Middle LTL n=177 (range: 1.10-1.45 kbp)		Longest LTL n=177 (range: 1.45-2.60 kbp)		
Age in years (mean, SD)	58.9	(6.9)	58.0	(7.1)	56.1	(6.9)	<0.001
Current smokers (%)	48.9		44.6		43.5		0.054
Heavy drinkers (%)	18.5		17.0		20.3		0.601
Married (%)	89.3		91.5		92.7		0.531
University education (%)	39.9		33.3		41.8		0.393
Systolic blood pressure (mean, SD)	140.7	(21.9)	142.0	(23.6)	141.2	(23.2)	0.861
Body mass index (mean, SD)	26.7	(4.0)	26.6	(4.1)	27.1	(4.3)	0.577
Total cholesterol cc. (mean, SD)	6.2	(1.1)	6.2	(1.2)	6.2	(1.0)	0.999
Self-reported history of prior CVD (%)	24.7		19.8		20.9		0.497

Note. SD – standard deviation; LTL – leukocyte telomere length; CVD – cardiovascular disease

[a] ANOVA or Chi-square test

Table S2 (supplementary material). Distribution of covariates across leucocyte telomere length tertiles among women

Covariates	Leucocyte telomere length tertiles						p-value[a]
	Shortest LTL n=204 (range: 0.26-1.20 kbp)		Middle LTL n=204 (range: 1.20-1.52 kbp)		Longest LTL n=204 (range: 1.52-4.08 kbp)		
Age in years (mean, SD)	58.2	(6.9)	58.0	(6.6)	55.9	(6.5)	<0.001
Current smokers (%)	7.4		6.9		7.8		0.552
Heavy drinkers (%)	0.49		1.47		1.47		0.302
Married (%)	65.7		56.9		65.7		0.104
University education (%)	27.0		24.0		34.3		0.120
Systolic blood pressure (mean, SD)	140.2	(24.7)	141.6	(25.8)	135.6	(22.7)	0.032
Body mass index (mean, SD)	30.1	(5.1)	29.3	(5.3)	29.6	(5.6)	0.345
Total cholesterol cc. (mean, SD)	6.7	(1.3)	6.6	(1.3)	6.6	(1.1)	0.742
Self-reported history of prior CVD (%)	18.1		22.6		17.2		0.339

Note. SD – standard deviation; LTL – leukocyte telomere length; CVD – cardiovascular disease

[a] ANOVA or Chi-square test

<u>Table S3 (Supplementary material).</u> Relationship between leucocyte telomere length tertiles and non-fatal CHD and stroke outcomes in the Russian arm of the HAPIEE study

Cardiovascular outcome (non-fatal)	n event	model	Leucocyte telomere length tertiles						Per 1 SD increase in telomere length		
			Shortest LTL	Middle LTL		Longest LTL					
			HR	HR	(95% CI)	HR	(95% CI)	p-value for trend	HR	(95%CI)	p-value
CHD	88	model 1	1.00 (ref)	0.88	(0.52-1.47)	0.96	(0.57-1.59)	0.865	0.90	(0.72-1.12)	0.352
		model 2	1.00 (ref)	0.82	(0.49-1.39)	1.02	(0.61-1.70)	0.968	0.92	(0.73-1.14)	0.442
Stroke	44	model 1	1.00 (ref)	0.51	(0.24-1.06)	0.63	(0.31-1.29)	0.178	0.82	(0.59-1.14)	0.243
		model 2	1.00 (ref)	0.49	(0.23-1.03)	0.65	(0.32-1.32)	0.193	0.83	(0.60-1.15)	0.263
CHD and stroke	124	model 1	1.00 (ref)	0.81	(0.52-1.25)	0.93	(0.60-1.42)	0.723	0.92	(0.76-1.10)	0.362
		model 2	1.00 (ref)	0.76	(0.49-1.18)	0.96	(0.62-1.47)	0.836	0.93	(0.77-1.12)	0.431

Note. LTL – leukocyte telomere length; HR – hazard ratio; CI – confidence interval; SD – standard deviation; CHD – coronary heart disease;
model 1: adjusted for age and sex
model 2: adjusted for age, sex, smoking, alcohol, education, marital status, body mass index, systolic blood pressure, total cholesterol cc. and self-reported history of prior CVD
